# Higher Expression Levels of Aquaporin Family of Proteins in the Kidneys of Arid-Desert Living *Lepus yarkandensis*

**DOI:** 10.3389/fphys.2019.01172

**Published:** 2019-09-12

**Authors:** Jianping Zhang, Shuwei Li, Fang Deng, Buheliqihan Baikeli, Shuguang Huang, Binyu Wang, Guoquan Liu

**Affiliations:** ^1^College of Life Science, Tarim University, Alar, China; ^2^Department of Basic Veterinary Medicine, College of Animal Science and Veterinary Medicine, Huazhong Agricultural University, Wuhan, China; ^3^Key Laboratory of Protection and Utilization of Biological Resources in Tarim Basin, Tarim University, Alar, China; ^4^Anhui Province Key Laboratory of Translational Cancer Research, Department of Biochemistry, College of Laboratory Medicine, Bengbu Medical College, Bengbu, China

**Keywords:** aquaporin family proteins, kidneys, water transport, arid-desert living, *Lepus yarkandensis*

## Abstract

*Lepus yarkandensis* specifically lives in arid climate with rare precipitation of Tarim Basin in western China. Aquaporins (AQPs) are a family of channel proteins that facilitate water transportation across cell membranes. Kidney AQPs play vital roles in renal tubule water permeability and maintenance of body water homeostasis. This study aimed to investigate whether kidney AQPs exhibit higher expression in arid-desert living animals. Immunohistochemistry results revealed localization of AQP1 to the capillary endothelial cells in glomerulus and epithelial cells in proximal tubule and descending thin limbs, AQP2 to the apical plasma membrane of principal cells in the cortical collecting duct (CCD), outer medullary collecting duct (OMCD), and IMCD cells in the initial inner medullary collecting duct (IMCD1) and middle IMCD (IMCD2), and AQP3 and AQP4 to the basolateral plasma membrane of principal cells and IMCD cells in CCD, OMCD, IMCD1, and IMCD2 in *L. yarkandensis* kidneys. Quantitative real-time PCR analysis showed higher mRNA levels of *AQP1*, *AQP2*, *AQP3*, and *AQP4* in *L. yarkandensis* kidneys compared with *Oryctolagus cuniculus*. Similar results were obtained by western blotting. Our results suggested that higher expression levels of AQP1, AQP2, AQP3, and AQP4 in *L. yarkandensis* kidneys favored for drawing more water from the tubular fluid.

## Introduction

*Lepus yarkandensis* (Yarkand hare) belong to *Lepus*, Leporidae, and Lagomorphs, and is an endemic species that live specifically in the Tarim Basin of western China. Their living environment is characterized by extremely dry continental climate. The annual precipitation is less than 100 mm, mostly below 50 mm, and the precipitation is scarce, as the evaporation is strong with about 39°C in summer, the air remained very dry (Li et al., 2006). Due to long-term living of *L. yarkandensis* in the environment of drought and water shortage, its size remained smaller and it is the smallest individual among all other hares in China. The content of Na^+^ is higher and Ca^2+^ is lower in blood of *L. yarkandensis* when compared to that of *O. cuniculus*, suggesting a strong adjusting ability of *L. yarkandensis* in maintaining the body water (Gao et al., 1998). In recent years, several researchers have investigated genetic variation, population structure and phylogeography of Yarkand hare (Li et al., 2006), the matrilineal and demographical histories of Tarim Basin’s endemic Yarkand hare (Shan et al., 2011), the bidirectional introgressive hybridization between *L. capensis* and *L. yarkandensis* (Wu et al., 2011), for the presence of Leishmania in *L. yarkandensis* (Gao et al., 2015), and genetic diversity in male-specific SRY gene of *L. yarkandensis* (Wu et al., 2010). So, the body of *L. yarkandensis* has specialized height, and it has strong drought tolerance ability when compared with rabbits and other hares (Li, 2011; Hui and Zhao, 2013; Yu and Zhang, 2013; Zhang et al., 2015, 2016). However, little information is available regarding the mechanisms and determinants of drought tolerance in *L. yarkandensis*.

Aquaporins (AQPs) are a family of highly conserved membrane-channel proteins (King et al., 2004) that facilitate water transportation across cell membranes (King and Agre, 1996), and play a significant role in water homeostasis and osmoregulation (Agre and Kozono, 2003). The kidneys play a central role in the regulation of body salt and water balance. The human kidney, which contains, on average, about 1 million nephrons, and each nephron segment has a well-defined water permeability that is associated with the presence or absence of different AQPs (Nielsen et al., 2002). Multiple AQP subtypes are expressed in the kidneys (King et al., 2004; Fenton and Knepper, 2007). AQP1 is extremely abundant in the apical membrane of epithelial cells in the proximal tubule and descending thin-limb, and the endothelial cells in the descending vasa recta (Nielsen et al., 1993b, 1995). AQP2 is exclusively present in the apical membrane of the collecting duct (CD) principal cells and inner medullary CD (IMCD) cells (Nielsen et al., 1993a). AQP3 is found in the basolateral membrane of CD principal cells in the cortex and the outer medulla regions (Ecelbarger et al., 1995; Ishibashi et al., 1997; Coleman et al., 2000). AQP4 is expressed in the basolateral membrane of principal cells in the outer medullary CD and IMCD (Terris et al., 1995). AQP6 is present in the intracellular vesicles in the CD intercalated cells (Yasui et al., 1999). AQP7 and AQP8 are expressed in the brush borders of proximal tubular epithelium (Ishibashi et al., 2000; Nejsum et al., 2000; Elkjaer et al., 2001; Nielsen et al., 2002).

In the kidneys, the final water reabsorption takes place in the CD epithelium (Clapp et al., 1989; Fenton and Knepper, 2007). The transepithelial water transport is a transcellular route, where the water is reabsorbed apically via AQP2 and exits basolaterally via AQP3 and AQP4, producing water from the renal tubular lumen to the interstitium and concentrated urine (Nielsen et al., 2002). There are several evidences that AQPs play a role in renal water permeability and urinary concentration (Verkman, 2008a, b). Mice lacking AQP1 or AQP2 or AQP3 or AQP4 manifest defects in urinary concentrating ability to different extents. Deletion of AQP1 in mice resulted in remarkable occurrence of polyuria (Ma et al., 1998). AQP3 null mice are able to concentrate their urine partially to approximately 30% of wild-type mice, and the double-knockout of AQP3 and AQP4 in mice presented impairment of urinary-concentrating capacity when compared with AQP3 single-knockout mice (Ma et al., 2000). Mutations in AQP2 water channel caused rare genetic disorder of nephrogenic diabetes insipidus (NDI), resulting in the excretion of large volumes of dilute urine (Deen et al., 1994). Mechanistic studies have confirmed the involvement of AQP1 in near-isoosmolar fluid absorption in the proximal tubule, and in countercurrent multiplication and exchange mechanisms to produce medullary hypertonicity in the antidiuretic kidney. Deletion of AQP2, AQP3 and AQP4 impaired urinary concentrating ability by reducing the transcellular water permeability in the CD. Animal study revealed that the decreased expression of AQP1, AQP2, AQP3, and AQP4 reduced the renal function. AQP1, AQP2, AQP3, and AQP4 are thus required for renal water permeability and formation of concentrated urine.

*Lepus yarkandensis* is distributed only in the drought area of Tarim Basin of China and therefore it had specialized morphological traits for their adaptations, but the molecular mechanisms for their survival in extreme environments are still unknown. Water is precious to the animals living in arid-deserts, and efficient use of limited water resources determines their survival. Renal AQPs play a significant role in water permeability and urinary concentration in renal tubules, maintaining body water. In the present study, we examined the histological structure and the expression of AQP1, AQP2, AQP3, and AQP4 in *L. yarkandensis* kidneys in comparison with *O. cuniculus*. The results will help us to understand the role of AQPs in the adaptation of *L. yarkandensis* to arid environment, and also to understand more about the molecular mechanisms of survival and adaptation in arid environments of arid-desert living animals.

## Materials and Methods

### Experimental Animals and Tissue Collection

In the study, adult male rabbits were used. 10 *L. yarkandensis* were collected from Tarim basin, and were confirmed as adults based on the skull length of over 75.50 mm. And 10 healthy adult *O. cuniculus* were purchased from animal laboratory station of tarim university. The health status of the animal remained good. *L. yarkandensis* was not reared in laboratory, it tested directly, and *O. cuniculus* had free access to water and rabbit chow in laboratory before experiment. Blood samples were collected from these animals for determination of hematological and biochemical parameters. The animals were then intravenously injected with 20% urethane (Sigma-Aldrich, Shanghai, China) with 0.4 ml/kg weight. Both kidneys were rapidly removed and washed briefly in isotonic saline. The length, width and thickness of the right kidney and the thickness of the cortex and the medulla were measured by vernier caliper. And the right renal medulla was removed for analyzing the kidney protein and the RNA. Then the left kidneys were dissected partially to obtain the cortex, outer medulla and inner medulla, infused with 4% paraformaldehyde (Sigma-Aldrich, Shanghai, China) and then fixed overnight for histologic examination. Moreover, the tissue was used from *O. cuniculus* before and after being deprived of water for 36 h (*n* = 6 for each group) (Ma et al., 1998).

### Hematoxylin and Eosin (HE)

The kidney tissues of *L. yarkandensis* were fixed in 4% paraformaldehyde. For histological analysis, the tissues after embedding in paraffin were cut into 6 μm thick sections, and then stained using hematoxylin and eosin (Sigma-Aldrich, Shanghai, China).

### Immunohistochemistry

AQP1, AQP2, AQP3, and AQP4 were evaluated in fixed left renal cortex, outer medulla, and inner medulla of *O. cuniculus* and *L. yarkandensis* by immunohistochemical staining. Fixed cortex, outer medulla and inner medulla were washed in phosphate-buffered saline (PBS), and then were separated for paraffin embedding. Paraffin-embedded cortex, outer medulla and inner medulla were sectioned at 6 μm for immunohistochemical staining of AQP1, AQP2, AQP3, and AQP4, and were deparaffinized by washing in xylene three times for 10 min each. This was followed by rehydration through a series of ethanol washes from 100% to 70% ethanol. The slides were then placed in methanol containing 0.5% hydrogen peroxide for removing the endogenous peroxidase activity. Non-specific binding was blocked by incubating the slides for 1 h at room temperature in 5% BSA. The cortex, outer medulla and the inner medulla sections were incubated with antibodies against AQP1, AQP2, AQP3, and AQP4 (at dilutions of 2.0 or 4.0 or 4.0 or 4.0 μg/ml) (Proteintech, Wuhan, China) overnight at 4°C. The sections were rinsed in 0.1 M PBS (PH 7.2–7.4) and then incubated for 30 min at room temperature with HRP-labeled goat anti-rabbit secondary antibody (Proteintech, Wuhan, China). The sections were washed with PBS, incubated with diaminobenzidine for 6 min and then washed again. The tissue sections were stained using hematoxylin (Sigma-Aldrich, Shanghai, China) for 40 s and then washed under running water for 5 min. Besides, the primary antibody was substituted with PBS, and the controls underwent similar procedure as described before. The immunostained sections were observed and photographed on a microscope (Motic BA600-4, Beijing, China). The captured images were analyzed in the IpWin32 software. IpWin32 software was used to analyze the integrated optical density (IOD) and area of immunopositive cells in each group, and the difference in the average density (IOD/Area) between the groups was compared.

### Quantitative Real-Time PCR

Reverse-transcribed cDNA products were amplified by polymerase chain reaction (PCR) with primers specific for *AQP1*, *AQP2*, *AQP3*, *AQP4*, and β*-actin* (Table 1). PCR reactions consisted of 10 μM of each primer, dNTP Mixture, Mg^2+^, SYBR Green I, and Taq polymerase (TaKaRa) in a total reaction volume of 10 μl. Quantitative RT-PCR was performed in a LightCycler 480 (Roche) under the following conditions: 95°C for 30 s, then 40 cycles of 95°C for 10 s, 60°C for 20 s and 72°C for 20 s. The relative analysis of gene expression was evaluated using the 2^–ΔΔ^ ^Ct^ method. The values were corrected by quantitation of β*-actin* values and were expressed as gene/β*-actin* ratio.

**TABLE 1:** Sequences of primers for real-time PCR.

**Gene**	**Forward (5′–3′)**	**Reverse (5′–3′)**
β*-actin*	TTTTGAATGGTCAGCCATCGT	GAGACCAAAAGCCTTCATACATCTC
*AQP1*	GACTACACTGGCTGTGGCATTAAC	GATCCAGTGGTTGTTGAAGTTGTG
*AQP2*	TGTCATCACCGGCAAGTTTG	AGATCAGCGAGCCCAGGAT
*AQP3*	GGATCAAGCTGCCCATCTACA	CATCATAATATAGCCCGAAAACGA
*AQP4*	AAAACCAGGAGTGATGCATGTG	GGAAGACAACACCTCTCCAGATG

*AQP, aquaporin.*

### Western Blotting Analysis

Total proteins were isolated from the kidneys of *O. cuniculus* and *L. yarkandensis*. 100 mg right-kidney tissue was isolated and placed in chilled lysis buffer containing 0.04 M Tris–Hcl (PH 7.4), 0.82% NaCl, 1.5% Triton X-100, 0.5% deoxycholic acid sodium salt, 0.1% SDS, protease inhibitor cocktail (Sigma-Aldrich, Shanghai, China), and 1 mM PMSF. The tissues were homogenized in 1 ml of lysis buffer. The homogenates were placed on ice for 20 min, and then were centrifuged at 12,000 × g for 20 min at 4°C. The supernatants were then collected. The total protein concentration was measured using BCA protein assay reagent kit (Aidlab, Beijing, China) according to the manufacturer’s protocol.

The total proteins were solubilized in laemmli sample buffer at 70°C for 8 min, and then were subjected to SDS-polyacrylamide gel electrophoresis. After transferring to polyvinylidene difluoride (PVDF) membranes (Bio-Rad), western blotting membranes were stripped and blocked, followed by incubation for overnight at 4°C with antibodies against AQP1, AQP2, AQP3, and AQP4 or anti-β-actin antibody (Proteintech, Wuhan, China). After washing, the membranes were incubated with horseradish peroxidase (HRP)-labeled anti-rabbit secondary antibody (Proteintech) for 1 h at room temperature, and then were visualized via enhanced chemiluminescence (SuperSignal, Pierce). Western blotting was scanned using Tanon 5200 and then the labeling density was quantitated using Gel-Pro Analyzer4. The densitometry results of *L. yarkandensis* are reported as volume integrated values and expressed in fractions, and then compared with the mean values of *O. cuniculus*, normalized by β-actin protein.

### Statistical Analyses

Statistical analysis software Graphpad Prism was used to calculate the means (M) and Standard error of mean (Sem) and used M ± Sem for histogram. Student’s *t*-test was used to assess the differences between the two groups. A *P*-value of less than 0.05 was considered to be statistically significant, ^∗^*p* < 0.05; ^∗∗^*p* < 0.01; ^∗∗∗^*p* < 0.001; and ns, *p* > 0.05.

## Results

### Hematological and Biochemical Parameters of *O. cuniculus* and *L. yarkandensis* Blood

We tested the hematological and biochemical parameters in the blood samples of *O. cuniculus* and *L. yarkandensis* and found the higher levels of urea, sodium, potassium, and chlorine in the blood of *L. yarkandensis* than in the blood of *O. cuniculus* (*n* = 10 for each group, *P* < 0.01) (Table 2). In order to maintain balance, these substances maybe also higher levels in *L. yarkandensis* urine.

**TABLE 2:** Hematological and biochemical parameters of *O. cuniculus* and *L. yarkandensis* blood.

**Item**	***O. cuniculus***	***L. yarkandensis***
Potassium K/(mmol/L)	3.16 ± 0.38	8.28 ± 0.59^∗∗^
Sodium Na/(mmol/L)	140.50 ± 2.52	148.50 ± 0.71^∗∗^
Chlorine Cl/(mmol/L)	104.25 ± 2.22	117.18 ± 2.50^∗∗^
Calcium Ca/(mmol/L)	3.40 ± 0.23	2.92 ± 0.18
Total protein TP/(g/L)	51.77 ± 2.14	49.13 ± 1.86
Albumin ALB/(g/L)	36.03 ± 3.07	39.47 ± 1.94
Globulin GLB/(g/L)	15.73 ± 1.01	9.53 ± 1.57^∗^
White ball ratio A/G	2.33 ± 0.35	4.17 ± 0.45^∗^
Total bilirubin TBIL/(μmol/L)	0.55 ± 0.31	3.68 ± 1.21^∗^
Direct bilirubin DBIL/(μmol/L)	0.30 ± 0.08	0.85 ± 0.30^∗^
Indirect bilirubin IBIL/(μmol/L)	0.37 ± 0.23	2.93 ± 0.72^∗^
Alanine aminotransferase ALT/(U/L)	35.33 ± 5.69	247.33 ± 15.63^∗∗^
Aspartate aminotransferase AST/(U/L)	40.33 ± 18.82	299.67 ± 60.12^∗∗^
Aspartate/alanine	1.23 ± 0.75	1.20 ± 0.17
Alkaline phosphatase ALP/(U/L)	116.33 ± 29.14	78.67 ± 12.10
The transfer glutamylamino enzyme GGT/(U/L)	8.33 ± 1.53	20.67 ± 1.53^∗∗^
Total cholesterol CHOL/(mmol/L)	1.10 ± 0.19	0.81 ± 0.10^∗^
Triglycerides TG/(mmol/L)	0.66 ± 0.09	1.19 ± 0.23^∗∗^
Uric acid UA/(μmol/L)	10.23 ± 1.47	22.27 ± 3.73^∗^
Urea UN/(mmol/L)	6.07 ± 0.23	20.80 ± 2.51^∗∗^
Creatinine Cr/(μmol/L)	68.30 ± 10.42	104.00 ± 1.83^∗∗^
Glucose G/(mmol/L)	14.93 ± 1.51	4.73 ± 1.30^∗∗^
Lactate dehydrogenase LDH/(U/L)	159.75 ± 55.25	1422.0 ± 146.76^*⁣**^
Creatine kinase CK/(U/L)	728.67 ± 258.64	4484.0 ± 947.37^∗^

*^∗^*p* < 0.05; ^∗∗^*p* < 0.01; and ^∗∗∗^*p* < 0.001.*

### Histology of *L. yarkandensis* Kidneys

**FIGURE 1:**
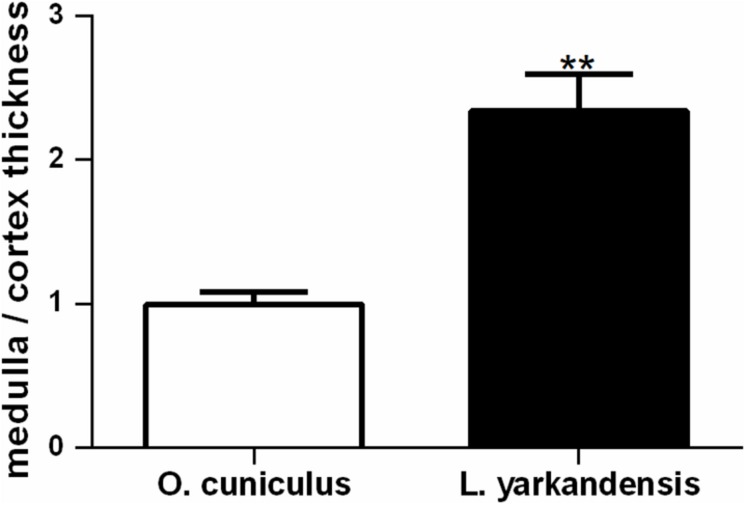
Wider medulla in *L. yarkandensis* kidneys. Medulla to cortex thickness ratios of kidneys were determined from *O. cuniculus* and *L. yarkandensis* (*n* = 6 for each group), ^∗∗^*p* < 0.01. The ratio of medulla to cortex thickness was higher in *L. yarkandensis* than that in *O. cuniculus*.

*Lepus yarkandensis* kidneys were bean-shaped, 27.00 ± 1.16 mm long, 14.33 ± 0.88 mm wide, and 10.67 ± 0.88 mm in thickness. The length ratio of kidney to body of *L. yarkandensis* and *O. cuniculus* was approximately 0.06. The weight of the kidney of *L. yarkandensis* and *O. cuniculus* were 0.018 ± 0.002 and 0.015 ± 0.003 kg, respectively. A transverse section of the kidney showed an outer cortex and an inner medulla. The thickness of *L. yarkandensis* cortex was approximately 2 mm, but the medulla was 7 10 mm in thickness. The thickness ratio of medulla to cortex of *L. yarkandensis* was (3.5 5.0):1. Similarly, the *O. cuniculus* cortex and medulla thickness were measured. The thickness of *O. cuniculus* cortex and medulla were approximately 2 mm and 3 4 mm in thickness, respectively. And the thickness ratio of medulla to cortex of *O. cuniculus* showed a ratio of (1.5 2.0):1. The ratio of medulla to cortex thickness was higher in *L. yarkandensis* than that in *O. cuniculus* (*P* < 0.01) (Figure 1).

**FIGURE 2:**
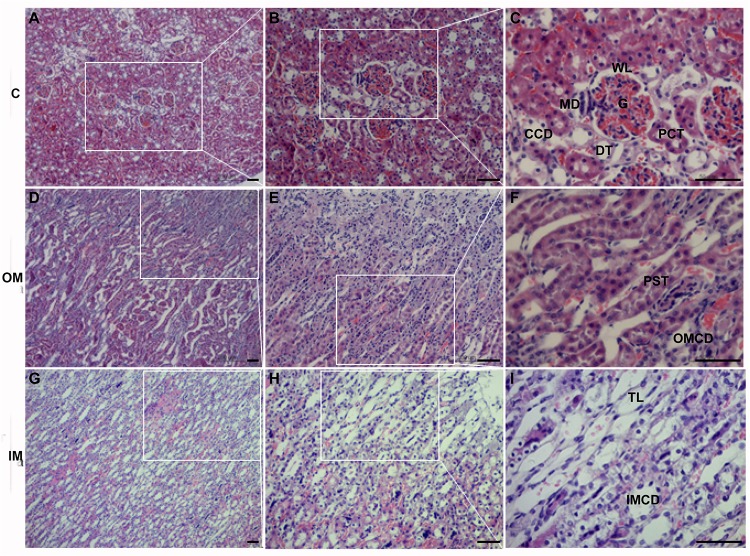
The histology of cortex **(A–C)**, outer medulla **(D–F)** and inner medulla **(G–I)** in *L. yarkandensis* kidneys (scale bar 200 μm, **A,D,G**; scale bar 100 μm, **B,E,H**; scale bar 50 μm, **C,F,I**). White boxes indicate enlarged local areas. cortex (C), outer medulla (OM), inner medulla (IM), wall layer (WL), macula densa (MD), glomerulus (G), cortical collecting duct (CCD), proximal convoluted tubule (PCT), distal tubule (DT), proximal straight tubule (PST), outer medullary collecting duct (OMCD), thin limbs (TL), inner medullary collecting duct (IMCD).

The cortex (C) of *L. yarkandensis* kidney was occupied by renal corpuscle (Figure 2A), which consisted of glomerulus and renal capsule (Figure 2B). The shape of the glomerulus (G) was round or oval, the wall layer (WL) of the renal capsule consisted of a single layer of flat epithelium, and there were many renal tubules around the renal corpuscle, including the proximal convoluted tubule (PCT), the distal tubule (DT) and the cortical collecting duct (CCD) (Figure 2C). The vascular pole of the renal corpuscle, where the juxtaglomerular complex (JGC) was present, was composed of juxtaglomerular cell, macula densa (MD) and extraglomerular mesangial cell (Figure 2C).

The renal medulla of *L. yarkandensis* was demarcated into outer medulla (OM) and inner medulla (IM). The narrow straight columns of the medullary rays contained proximal straight tubule (PST) and outer medullary CD (OMCD) (Figures 2D–F). With the help of light microscopy, we observed that the *L. yarkandensis* had thin limbs, and were longer due to the extension of descending thin limbs (TL) to the IM (Figures 2G–I). The CD along the cortical labyrinth toward the medullary rays descended toward the renal papilla, forming a larger diameter CD called inner medullary collecting duct (IMCD) (Figures 2G–I). The CD can be divided into CCD, OMCD, and IMCD based on its location. From the cortex to the medulla, the diameter of the CD was larger and the shape of the CD principal cells remained higher, becoming high columnar cell in IM, and called as IMCD cell (Clapp et al., 1989).

### Localization of AQP1, AQP2, AQP3, and AQP4 in *L. yarkandensis* Kidneys

**FIGURE 3:**
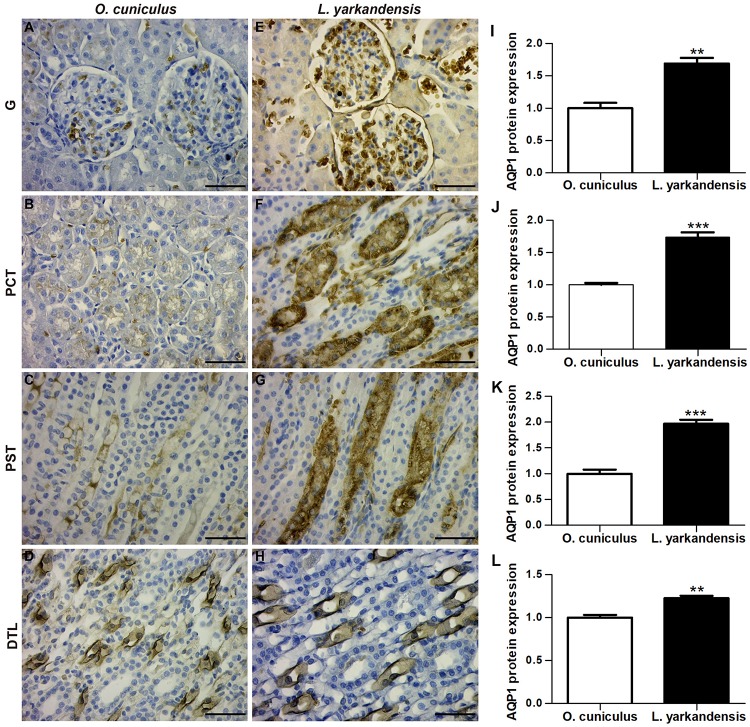
AQP1 protein distribution in renal G, PCT, PST, and DTL in a tissue section of *O. cuniculus* **(A–D)** and *L. yarkandensis* **(E–H)**. **(A–H)** Paraffin sections (6 μm) of renal cortex and medulla of *O. cuniculus* and *L. yarkandensis*. The sections were incubated with anti-AQP1 antibody scale bar 50 μm. **(I–L)** Densitometry of all immunohistochemistry results of renal G and PCT and PST and DTL from *O. cuniculus* and *L. yarkandensis* (*n* = 6 for each group), ^∗∗^*p* < 0.01 and ^∗∗∗^*p* < 0.001. In *O. cuniculus*, weak labeling was detected in G, PCT, PST, and DTL cells **(A–D)**. In contrast, strong labeling was detected in G, PCT, PST, and DTL cells **(E–H)** of *L. yarkandensis*. Average density showed a significant higher expression of AQP1 protein abundance in G, PCT, PST, and DTL of *L. yarkandensis* compared to that of *O. cuniculus* **(I–L)**.

AQPs are a family of highly conserved membrane-channel proteins (King et al., 2004). AQP1, AQP2, AQP3, and AQP4 mRNA and amino acid sequences were available in GenBank (Table 3). Furthermore, amino acid sequence alignment revealed that the identity of AQP1 amino acid sequence among *O. cuniculus* and *L. yarkandensis* to be 99%, and the epitope identity of AQP1 antibody and AQP1 of *O. cuniculus* was 96%. The alignment results of AQP2, AQP3, and AQP4 were similar to AQP1. Thus, we compared the differences in protein expression in the samples from *O. cuniculus* and *L. yarkandensis* based on immunohistochemistry and western blotting results. Immunohistochemistry was performed to analyze protein localization of AQP1, AQP2, AQP3, and AQP4 in the renal cortex and medulla of *O. cuniculus* and *L. yarkandensis*. AQP1 staining was localized to the capillary endothelial cells in G and epithelial cells in PCT and PST (Figures 3A–H). In *O. cuniculus*, weak labeling was detected in G, PCT, PST, and DTL cells (Figures 3A–D). In contrast, strong labeling was detected in G, PCT, PST, and DTL cells of *L. yarkandensis* (Figures 3E–H). Average density of immunohistochemistry results indicated a higher expression of AQP1 levels in G, PCT, PST, and DTL cells of *L. yarkandensis* compared to that of *O. cuniculus* (*P* < 0.01), especially in PCT and PST cells (*P* < 0.001) (Figures 3I–L).

**TABLE 3:** GenBank accession numbers for *O. cuniculus* and *L. yarkandensis* nucleotide sequences.

**Gene**	**Accession number**	
	***O. cuniculus***	***L. yarkandensis***
*AQP1*	XM_017344416	MK947034
*AQP2*	XM_002711130	MK947035
*AQP3*	XM_002708029	MK947036
*AQP4*	XM_002713474	MK947038

*AQP, aquaporin.*

**FIGURE 4:**
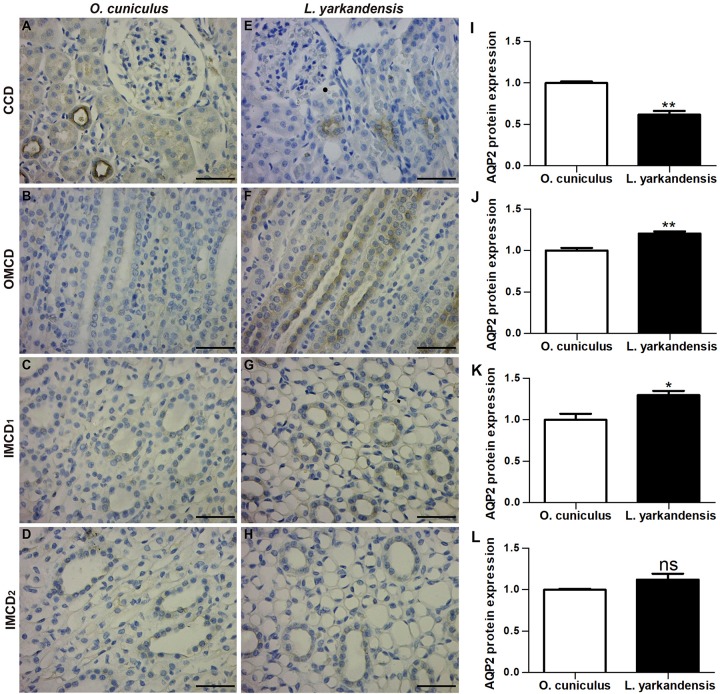
AQP2 protein distribution in renal CCD, OMCD, IMCD1, and IMCD2 in a tissue section of *O. cuniculus* **(A–D)** and *L. yarkandensis* **(E–H)**. **(A–H)** Paraffin sections (6 μm) of renal cortex and medulla of *O. cuniculus* and *L. yarkandensis*. The sections were incubated with anti-AQP2 antibody scale bar 50 μm. **(I–L)** Densitometry of all immunohistochemistry results of renal CCD, OMCD, IMCD1, and IMCD2 from *O. cuniculus* and *L. yarkandensis* (*n* = 6 for each group) ^∗^*p* < 0.05; ^∗∗^*p* < 0.01; and ns, *p* > 0.05. In *O. cuniculus*, strong labeling was detected in the apical plasma membranes of CDD principal cells **(A)**, whereas weak labeling was detected in the principal cells of OMCD, IMCD1, and IMCD2 **(B–D)**. In *L. yarkandensis*, weak labeling was detected in the apical plasma membranes of CDD principal cells **(E)**, prominent apical labeling was detected in OMCD **(F)** and IMCD1 **(G)**, and weak labeling was detected in IMCD2 **(H)**. Average density showed a dramatic higher expression of AQP2 protein abundance in OMCD and IMCD1, and a lower expression in CCD of *L. yarkandensis* compared to that of *O. cuniculus* **(I–L)**.

AQP2 staining was localized to the apical plasma membrane of principal cells in renal CCD, OMCD, the apical and basolateral plasma membranes of IMCD cells in the initial IMCD (IMCD1) and middle IMCD (IMCD2) of *O. cuniculus* and *L. yarkandensis* (Figures 4A–H). In *O. cuniculus*, light microscopy revealed strong labeling of AQP2 in the apical plasma membrane domains of CCD principal cells (Figure 4A), whereas weak labeling was detected in the principal cells of OMCD and IMCD cells of IMCD1 and IMCD2 (Figures 4B–D). In contrast, weak labeling was detected in the apical plasma membranes of CDD principal cells (Figure 4E), prominent apical labeling was detected in OMCD (Figure 4F) and IMCD1 (Figure 4G), and weak labeling was detected in IMCD2 (Figure 4H) of *L. yarkandensis*. Average density results of *L. yarkandensis* when compared with *O. cuniculus* demonstrated a higher expression of AQP2 levels in OMCD (*P* < 0.01) and IMCD1 (*P* < 0.05), and a lower expression in CCD (*P* < 0.01) (Figures 4I–L).

**FIGURE 5:**
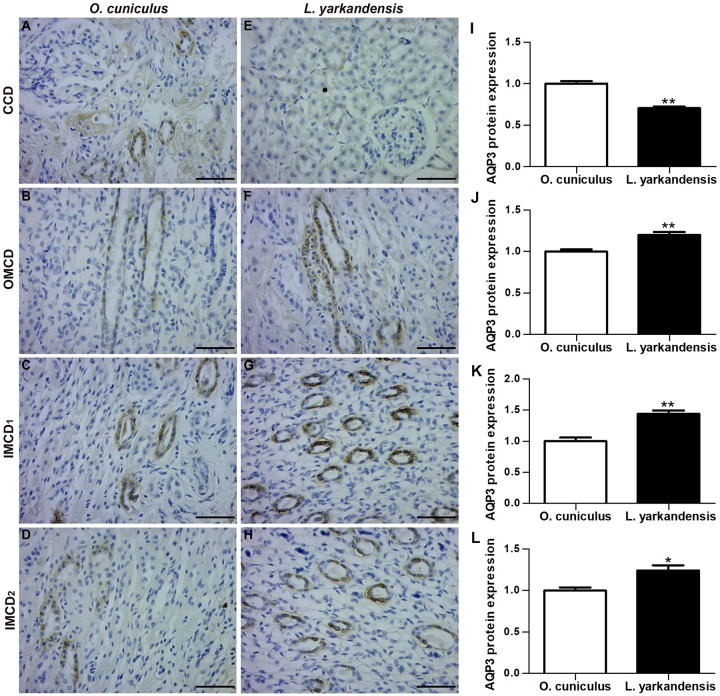
AQP3 protein distribution in renal CCD, OMCD, IMCD1, and IMCD2 in a tissue section of *O. cuniculus* **(A–D)** and *L. yarkandensis* **(E–H)**. **(A–H)** Paraffin sections (6 μm) of renal cortex and medulla of *O. cuniculus* and *L. yarkandensis*. The sections were incubated with anti-AQP3 antibody scale bar 50 μm. **(I–L)** Densitometry of all immunohistochemistry results of renal CCD, OMCD, IMCD1 and IMCD2 from *O. cuniculus* and *L. yarkandensis* (*n* = 6 for each group). ^∗^*p* < 0.05 and ^∗∗^*p* < 0.01. In *O. cuniculus*, strong labeling was detected in the basolateral plasma membranes of CDD principal cells **(A)**, whereas weak labeling was detected in the principal cells of OMCD, IMCD1, and IMCD2 **(B–D)**. In *L. yarkandensis*, weak labeling was detected in the basolateral plasma membranes of CDD principal cells **(E)**, more prominent basolateral labeling was detected in OMCD **(F)** and IMCD1 **(G)**, and weak labeling was detected in IMCD2 **(H)**. Average density showed a significant higher expression of AQP3 protein abundance in OMCD and IMCD1, and a lower expression in CCD of *L. yarkandensis* compared to that of *O. cuniculus* **(I–L)**.

AQP3 staining was localized to the basolateral plasma membrane of principal cells and IMCD cells in renal CCD, OMCD, IMCD1, and IMCD2 (Figures 5A–H). In *O. cuniculus*, strong labeling of AQP3 was detected in the basolateral plasma membrane domains of CCD principal cells (Figure 5A), whereas weak labeling was detected in the principal cells of OMCD, and IMCD cells in IMCD1 and IMCD2 (Figures 5B–D). In contrast, weak labeling was detected in the basolateral plasma membranes of CDD principal cells (Figure 5E), whereas obvious basolateral labeling was detected in OMCD (Figure 5F), IMCD1 (Figure 5G), and IMCD2 (Figure 5H) in *L. yarkandensis*. Average density results of *L. yarkandensis* when compared with *O. cuniculus* revealed a higher expression of AQP3 protein in OMCD and IMCD1 (*P* < 0.01), and a lower expression in CCD (*P* < 0.05) (Figures 5I–L).

**FIGURE 6:**
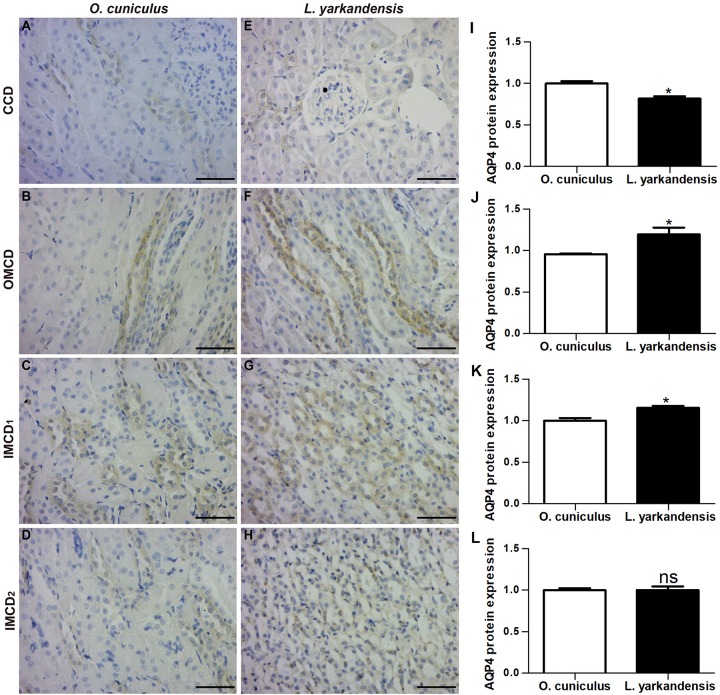
AQP4 protein distribution in renal CCD, OMCD, IMCD1, and IMCD2 in a tissue section of *O. cuniculus* **(A–D)** and *L. yarkandensis* **(E–H)**. **(A–H)** Paraffin sections (6 μm) of renal cortex and medulla of *O. cuniculus* and *L. yarkandensis*. The sections were incubated with anti-AQP4 antibody scale bar 50 μm. **(I–L)** Densitometry of all immunohistochemistry results of renal CCD, OMCD, IMCD1, and IMCD2 from *O. cuniculus* and *L. yarkandensis* (*n* = 6 for each group), ^∗^*p* < 0.05 and ns, *p* > 0.05. In *O. cuniculus*, labeling was detected in the basolateral plasma membranes of principal cells in CDD, OMCD, IMCD1, and IMCD2 **(A–D)**. In *L. yarkandensis*, weak labeling was detected in the basolateral plasma membranes of CDD principal cells **(E)**, more basolateral labeling was detected in OMCD **(F)** and IMCD1 **(G)**, and weak labeling was detected in IMCD2 **(H)**. Average density showed a higher expression of AQP4 protein abundance in OMCD and IMCD1, and a lower expression in CCD of *L. yarkandensis* compared to that of *O. cuniculus* **(I–L)**.

AQP4 was found in the basolateral plasma membrane of principal cells and IMCD cells in renal CCD, OMCD, IMCD1, and IMCD2 (Figures 6A–H). In *O. cuniculus*, labeling of AQP4 was detected in the basolateral plasma membrane of principal cells and IMCD cells in CCD, OMCD, IMCD1, and IMCD2 (Figures 6A–D). In *L. yarkandensis*, weak labeling was detected in the basolateral plasma membranes of CDD principal cells (Figure 6E), strong basolateral labeling was detected in OMCD (Figure 6F) and IMCD1 (Figure 6G), and weak labeling was detected in IMCD2 (Figure 6H). Average density results of *L. yarkandensis* when compared with *O. cuniculus* demonstrated a higher expression of AQP4 levels in OMCD and IMCD1 (*P* < 0.05), and a lower expression in CCD (*P* < 0.05) (Figures 6I–L). AQP1 or AQP2 or AQP3 or AQP4 antibody was substituted with PBS in the negative control, and the control slides included renal C, OM, and IM of a tissue section from *O. cuniculus* and *L. yarkandensis* in the immunohistochemistry experiment (Supplementary Figure S1). The control sections treated in the absence of primary antibody showed no positive staining.

### mRNA and Protein Expression of AQP1, AQP2, AQP3, and AQP4 in *L. yarkandensis* Kidneys

Immunohistochemistry data concluded that AQP1, AQP2, AQP3, and AQP4 proteins were higher in the renal medulla of *L. yarkandensis* than *O. cuniculus*. It is necessary to investigate whether the higher expression levels of AQP1, AQP2, AQP3, and AQP4 proteins abundance was in parallel with AQP1, AQP2, AQP3, and AQP4 mRNA and protein expressions in the renal medulla of *L. yarkandensis*. The nucleotide sequence alignment showed that the identity of AQP1 nucleotide sequence among *O. cuniculus* and *L. yarkandensis* was 99%, and primer-BLAST showed AQP1 primer that was specific to *O. cuniculus* AQP1. The alignment results of AQP2, AQP3 and AQP4 were similar to AQP1. So, we performed quantitative RT-PCR and western blotting to determine the levels of AQP1, AQP2, AQP3 and AQP4 mRNA and protein expression in the renal medulla of *O. cuniculus* and *L. yarkandensis*.

**FIGURE 7:**
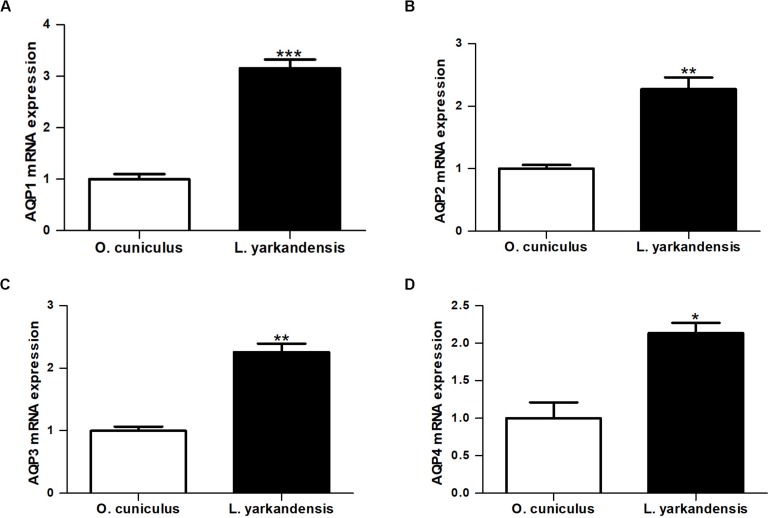
*AQP1*, *AQP2*, *AQP3*, and *AQP4* mRNA expression in the renal medulla of *O. cuniculus* and *L. yarkandensis*. Total RNAs were extracted from the renal medulla (*n* = 6 for each group), and 1.0 μg/samples were subjected to the RT reaction followed by PCR with primers specific for *AQP1*, *AQP2*, *AQP3*, and *AQP4*, and β*-actin*, respectively. The values were quantified as a ratio of the expression of each gene normalized for the expression level of β*-actin* for each sample as an internal loading control. Values were presented as fraction of the mean *O. cuniculus* values. *P*-values refer to the comparison of *L. yarkandensis* values with *O. cuniculus* values. **(A)** Quantitative RT-PCR analysis of *AQP1* mRNA in the renal medulla of *O. cuniculus* and *L. yarkandensis*. **(B)** Quantitative RT-PCR analysis of *AQP2* mRNA in the renal medulla of *O. cuniculus* and *L. yarkandensis*. **(C)** Quantitative RT-PCR analysis of *AQP3* mRNA in the renal medulla of *O. cuniculus* and *L. yarkandensis*. **(D)** Quantitative RT-PCR analysis of *AQP4* mRNA in the renal medulla of *O. cuniculus* and *L. yarkandensis*. Quantitative RT-PCR demonstrated that *AQP1*, *AQP2*, *AQP3*, and *AQP4* mRNA levels were higher in the renal medulla of *L. yarkandensis* than *O. cuniculus*. ^∗∗∗^*p* < 0.001, ^∗∗^*p* < 0.01, and ^∗^*p* < 0.05.

**FIGURE 8:**
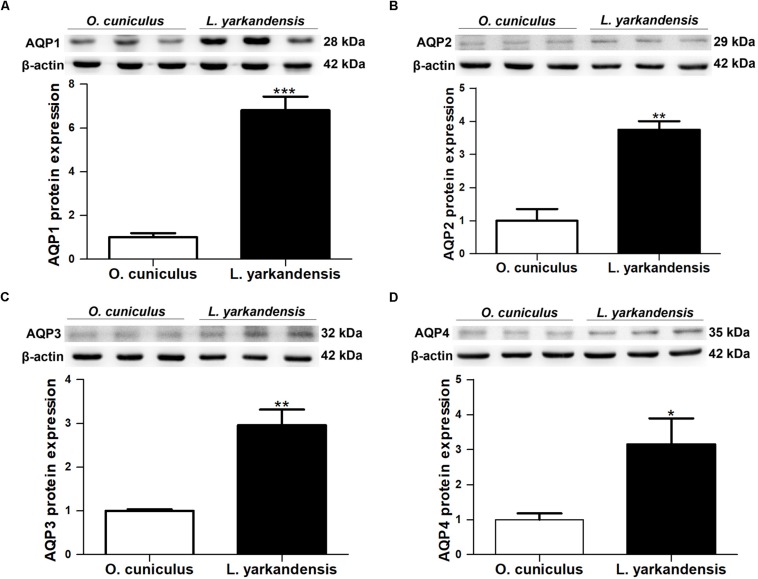
AQP1, AQP2, AQP3, and AQP4 proteins expression in renal medulla of *O. cuniculus* and *L. yarkandensis*. **(A)** (Top) Representative western blotting analyses of AQP1 protein expression in the renal medulla of *O. cuniculus* and *L. yarkandensis*. Immunoblots of total proteins were probed with anti-AQP1 antibody and identified 28 kDa bands, while β-actin antibody identified 42 kDa bands. (Bottom) Densitometry of all western blotting results of the renal medulla of *O. cuniculus* and *L. yarkandensis* (*n* = 6 for each group). **(B)** (Top) Representative western blotting analyses of AQP2 protein expression in the renal medulla of *O. cuniculus* and *L. yarkandensis*. (Bottom) Densitometry analysis of all western blotting results of the renal medulla of *O. cuniculus* and *L. yarkandensis*. **(C)** (Top) Representative western blotting analyses of AQP3 protein expression in the renal medulla of *O. cuniculus* and *L. yarkandensis.* (Bottom) Densitometry of all western blotting results of the renal medulla of *O. cuniculus* and *L. yarkandensis*. **(D)** (Top) Representative western blotting analyses of AQP4 protein expression in the renal medulla of *O. cuniculus* and *L. yarkandensis*. (Bottom) Densitometry analysis of all western blotting results of the renal medulla of *O. cuniculus* and *L. yarkandensis*; ^∗∗∗^*p* < 0.001, ^∗∗^*p* < 0.01, and ^∗^*p* < 0.05.

Quantitative RT-PCR demonstrated that *AQP1* mRNA levels were higher in the renal medulla of *L. yarkandensis* than *O. cuniculus* (*P* < 0.001) (Figure 7A). *AQP2* and *AQP3* mRNA levels were significantly raised in the renal medulla of *L. yarkandensis* (*P* < 0.01) (Figures 7B,C). *AQP4* mRNA levels were higher in the renal medulla of *L. yarkandensis* than *O. cuniculus* (*P* < 0.05) (Figure 7D). The levels of AQP1, AQP2, AQP3, and AQP4 proteins were detected by western blotting. Incubation with anti-AQP1, anti-AQP2, anti-AQP3, and anti-AQP4 antibodies demonstrated a band at 28, 29, 32, and 35 kDa, respectively (Figures 8A–D, Top), which was in agreement with the previously reported sizes of AQP1, AQP2, AQP3, and AQP4 proteins (Fernández-Llama et al., 1998). Densitometry of blotting results indicated a higher expression of AQP1 protein levels in the renal medulla of *L. yarkandensis* (*P* < 0.001) (Figure 8A, Bottom), showing a higher expression of AQP2 and AQP3 protein levels in the renal medulla of *L. yarkandensis* (*P* < 0.01) (Figures 8B,C, Bottom), and a higher expression of AQP4 protein levels in the renal medulla of *L. yarkandensis* (*P* < 0.05) (Figure 8D, Bottom). Together, these results indicated an increase of AQP1, AQP2, AQP3, and AQP4 at both mRNA and protein levels in the renal medulla of *L. yarkandensis*. Thus, these results were in concordance with immunohistochemistry results.

### Localization of AQP1, AQP2, AQP3, and AQP4 in *O. cuniculus* Renal Medulla Before and After Water Deprivation

**FIGURE 9:**
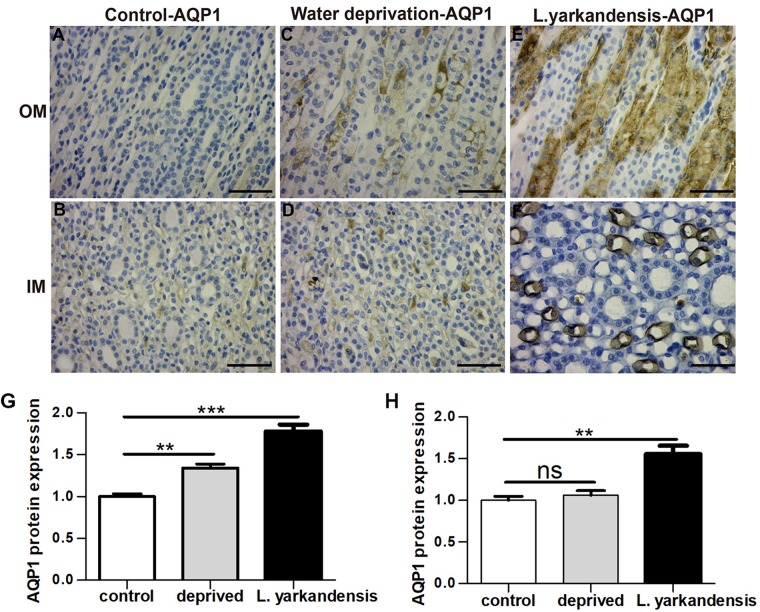
AQP1 protein distribution in renal OM and IM in a tissue section of a control *O. cuniculus*, a *O. cuniculus* subjected to 36 h of water-deprivation and *L. yarkandensis*. **(A–F)** Paraffin sections (6 μm) of renal medulla of *O. cuniculus*, water-deprived *O. cuniculus* and *L. yarkandensis*. The sections were incubated with anti-AQP1 antibody scale bar 50 μm. **(G,H)** Densitometry of all immunohistochemistry results of OM and IM from *O. cuniculus*, water-deprived *O. cuniculus* and *L. yarkandensis* (*n* = 6 for each group). ns, *p* > 0.05; ^∗∗^*p* < 0.01; and ^∗∗∗^*p* < 0.001. In the control *O. cuniculus*, weak labeling of AQP1 was detected in renal OM and IM **(A,B)**, whereas more labeling was observed in the water-deprived *O. cuniculus* **(C,D)**, and higher AQP1 labeling in *L. yarkandensis* **(E,F)**. Average density of the water-deprived *O. cuniculus* when compared with the control *O. cuniculus* revealed an increase in AQP1 levels in OMCD, but *L. yarkandensis* exhibited higher expression in OMCD and IMCD **(G,H)**.

**FIGURE 10:**
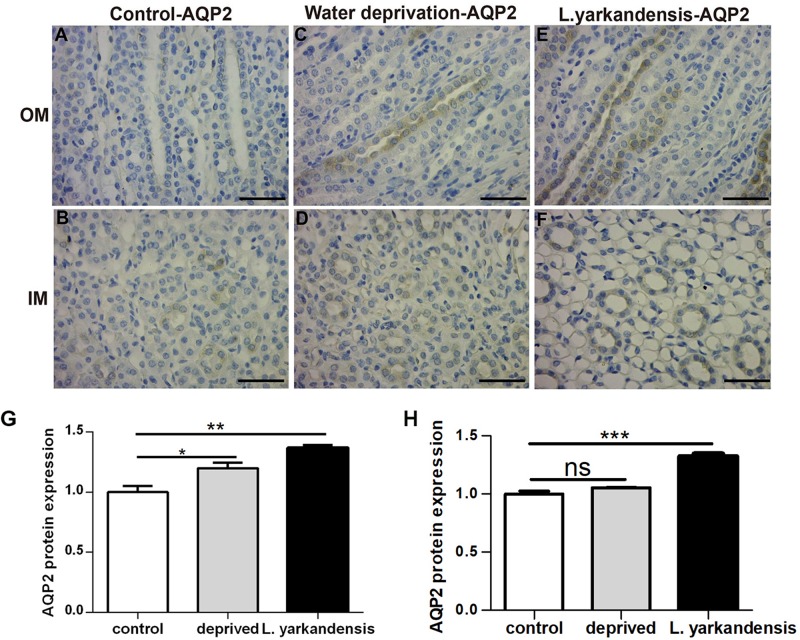
AQP2 protein distribution in renal OM and IM in a tissue section of a control *O. cuniculus*, a *O. cuniculus* subjected to 36 h of water-deprivation and *L. yarkandensis*. **(A–F)** Paraffin sections (6 μm) of renal medulla of *O. cuniculus*, water-deprived *O. cuniculus* and *L. yarkandensis*. The sections were incubated with anti-AQP2 antibody scale bar 50 μm. **(G,H)** Densitometry of all immunohistochemistry results of OM and IM from *O. cuniculus*, water-deprived *O. cuniculus* and *L. yarkandensis* (*n* = 6 for each group). ns, *p* > 0.05; ^∗^*p* < 0.05; ^∗∗^*p* < 0.01; and ^∗∗∗^*p* < 0.001. In the control *O. cuniculus*, weak labeling of AQP2 was detected in renal OM and IM **(A,B)**, whereas more labeling was observed in the water-deprived *O. cuniculus* **(C)**, and higher AQP2 labeling in *L. yarkandensis* **(E,F)**. Average density of the water-deprived *O. cuniculus* when compared with the control *O. cuniculus* revealed an increase in AQP2 levels in OMCD, but *L. yarkandensis* exhibited higher expression **(G,H)**.

**FIGURE 11:**
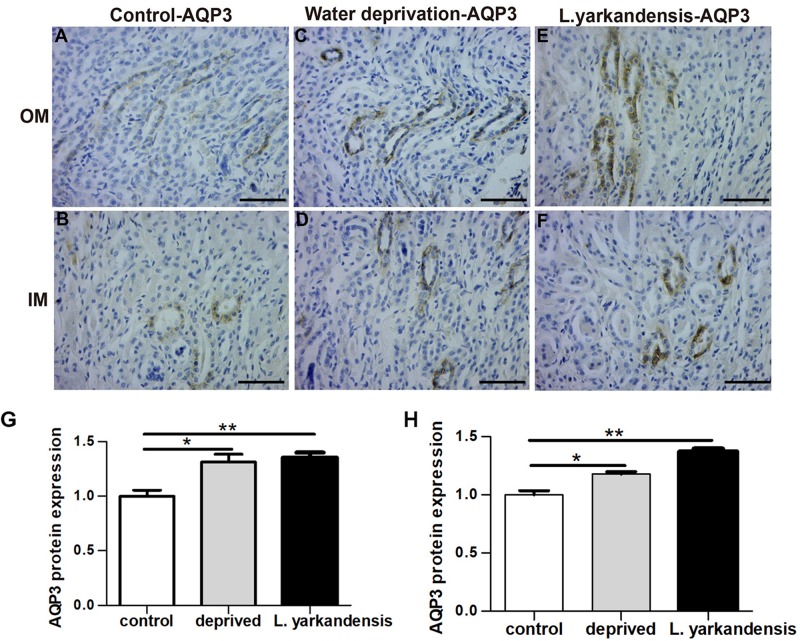
AQP3 protein distribution in renal OM and IM in a tissue section of a control *O. cuniculus*, a *O. cuniculus* subjected to 36 h of water-deprivation and *L. yarkandensis*. **(A–F)** Paraffin sections (6 μm) of renal medulla of *O. cuniculus*, water-deprived *O. cuniculus* and *L. yarkandensis*. The sections were incubated with anti-AQP3 antibody scale bar 50 μm. **(G,H)** Densitometry of all immunohistochemistry results of OM and IM from *O. cuniculus*, water-deprived *O. cuniculus* and *L. yarkandensis* (*n* = 6 for each group), ^∗^*p* < 0.05 and ^∗∗^*p* < 0.01. In the control *O. cuniculus*, weak labeling of AQP3 was detected in renal OM and IM **(A,B)**, whereas more labeling was observed in the water-deprived *O. cuniculus* **(C,D)**, and higher AQP3 labeling in *L. yarkandensis* **(E,F)**. Average density of the water-deprived *O. cuniculus* when compared with the control *O. cuniculus* revealed an increase in AQP3 level in OMCD and IMCD **(G,H)**.

**FIGURE 12:**
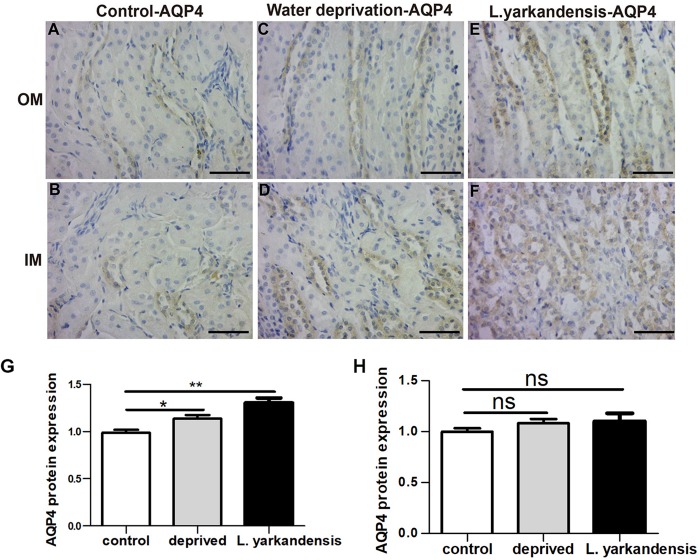
AQP4 protein distribution in renal OM and IM in a tissue section of a control *O. cuniculus*, a *O. cuniculus* subjected to 36 h of water-deprivation and *L. yarkandensis*. **(A–F)** Paraffin sections (6 μm) of renal medulla of *O. cuniculus*, water-deprived *O. cuniculus* and *L. yarkandensis*. The sections were incubated with anti-AQP4 antibody scale bar 50 μm. **(G,H)** Densitometry of all immunohistochemistry results of OM and IM from *O. cuniculus*, water-deprived *O. cuniculus* and *L. yarkandensis* (*n* = 6 for each group). ns, *p* > 0.05; ^∗^*p* < 0.05; and ^∗∗^*p* < 0.01. In the control *O. cuniculus*, weak labeling of AQP4 was detected in renal OM **(A)**, more labeling was observed in the water-deprived *O. cuniculus* **(C)**, and higher AQP4 labeling in *L. yarkandensis* **(E)**, whereas AQP4 labeling in the renal IM was no difference **(B,D,F)**. Average density of the water-deprived *O. cuniculus* when compared with the control *O. cuniculus* revealed an increase in AQP4 levels in OMCD **(G)** and no difference in IMCD **(H)**.

To investigate whether the higher expression levels of AQPs in the renal medulla occurs as a result of water shortage, we examined AQP1, AQP2, AQP3, and AQP4 expression in the renal medulla sections of *O. cuniculus* before and after being deprived of water for 36 h. In the control *O. cuniculus*, weak labeling of AQP1 was detected in renal OM and IM (Figures 9A,B), whereas more labeling was observed in the water-deprived *O. cuniculus* (Figures 9C,D), and higher AQP1 labeling in *L. yarkandensis* (Figures 9E,F). Average density of the water-deprived *O. cuniculus* when compared with the control *O. cuniculus* revealed a significant increase in AQP1 level in OMCD (*P* < 0.01), but *L. yarkandensis* exhibited higher expression in OMCD (*P* < 0.001) and IMCD (*P* < 0.01) (Figures 9G,H). The AQP2 labeling was similar to AQP1 (Figures 10A–H). The AQP3 and AQP4 labeling were detected in renal OM and IM (Figures 11A–F, 12A–F). Average density of the water-deprived *O. cuniculus* when compared with the control *O. cuniculus* revealed an increase in AQP3 level in OMCD and IMCD (*P* < 0.05) (Figures 11G,H), and AQP4 level in OMCD (*P* < 0.05) (Figure 12G) and no difference in IMCD (*P* > 0.05) (Figure 12H). The results showed higher expression levels of AQP proteins in the water-deprived *O. cuniculus.*

## Discussion

The anatomical structure of the kidneys in *L. yarkandensis* revealed wider medulla, and the results were in agreement with Mbassa study (Mbassa, 1988). The study concluded that animals living in desert or arid environments have wider renal medulla. Our finding was also consistent with a previous study, following a prolonged dehydration, the kidneys of desert rodents occurred morphological changes, and which is a selective hypertrophy (Sahni et al., 2011; Elgot et al., 2017). The relative thickness of the renal medulla in mammals reflected their adaptability to arid environments, as animals with a relatively wider renal medulla produced concentrated urine (Schmidt-Nielsen and O’dell, 1961). In addition, light microscopy illustrated that the thin limbs of *L. yarkandensis* kidney were longer, which was in line with wider medulla and higher ratio of medulla to cortex thickness, favoring the long loop of Henle. This causes high interstitial urea and salt concentrations at the tip of the papillae, driving water movement from the tubular fluid to the interstitial space. Moreover, our results implied that the JGC components of *L. yarkandensis* kidney are topographically intimate, and were consistent with the results of Moussa (2010) study in camel. The results suggested that the more intimate the JGC components are, the more effective will be the tubuloglomerular autoregulation of renal blood flow. Therefore, the wider medulla and long loop of Henle of *L. yarkandensis* kidneys provided a structural basis for the formation of high osmotic gradient in the medulla.

Then, we detected the expression of water channels (AQPs), which help in the transportation of water driven by osmotic gradient. Immunohistochemical analysis showed that AQP1 was localized to the apical and basolateral plasma membranes of G, PCT, PST, and DTL cells, AQP2 was localized to the apical plasma membrane of principal cells in CCD, OMCD, and the apical and basolateral plasma membranes of IMCD cells in IMCD1 and IMCD2, and AQP3 and AQP4 were localized to the basolateral plasma membrane of principal cells and IMCD cells in CCD, OMCD, IMCD1, and IMCD2. The distribution of AQP1, AQP2, AQP3, and AQP4 determined the water permeability of different nephron segments (King et al., 2004). The renal corpuscles are responsible for the formation of ultrafiltrate, where the absorption and the secretion of some materials take place as it passes along the renal tubule to form the urine. Blood is filtered at G and this filtrate then passes into the lumen of the renal tubule and then the CD. This filtrate was resorbed by AQP1 and other transporters that are expressed in the epithelial cells of PT and DTL. The final volume and the urine concentration were determined by CD AQPs that contained AQP2 of the apical membrane, and AQP3 and AQP4 of the basolateral membrane in the principal cells and IMCD cells (Poulsen et al., 2013). Average density of immunohistochemistry results indicated that AQP1, AQP2, AQP3, and AQP4 protein abundance was higher in the renal medulla of *L. yarkandensis* than *O. cuniculus*. Our study is the first to demonstrate AQP1 protein distribution and abundance in G, PCT, PST, and DTL, as well as AQP2, AQP3, and AQP4 protein abundance in CCD, OMCD, IMCD1, and IMCD2 in the kidneys of *L. yarkandensis*.

The expression of AQP1, AQP2, AQP3, and AQP4 at both mRNA and protein levels was higher in *L. yarkandensis* kidneys. Quantitative RT-PCR analysis showed higher mRNA levels of *AQP1*, *AQP2*, *AQP3*, and *AQP4* in *L. yarkandensis* renal medulla compared to those of *O. cuniculus* kidneys. Western blotting studies revealed a higher level of AQP1, AQP2, AQP3, and AQP4 proteins in *L. yarkandensis* renal medulla as well. Kortenoeven and Fenton (2014) found that water restriction and hypertonicity stimulation induced upregulation of renal AQP levels. AQP1 is present in the membranes of PT and DTL cells and its mRNA and protein levels are upregulated by three-fold by hypertonicity due to NaCl (Bouley et al., 2009). In addition, AQP2, AQP3, and AQP4 are present in the membranes of principal cells and IMCD cells in CD. AQP2 protein levels are increased in response to both arginine vasopressin and water deprivation (Terris et al., 1996). AQP3 expression is induced by dehydration at both mRNA and protein levels, and its protein level is increased due to its transcriptional level (Ishibashi et al., 1997). *AQP4* mRNA levels in renal medulla were increased in response to water restriction (Murillo-Carretero et al., 1999). Our results revealed higher expression of AQP1, AQP2, AQP3, and AQP4 levels in the renal medulla of *L. yarkandensis* compared with *O. cuniculus*. *L. yarkandensis* lives in an environment where water is scarce, and its diet consists of high salt. These conditions are similar to that of water restriction and hypertonicity, and we speculated that the levels of *L. yarkandensis* renal AQPs regulated their adaption. Taken together, these results revealed that the expression of AQP2, AQP3, and AQP 4 was regulated in a coordinated manner.

Immunohistochemical, quantitative RT-PCR, and western blotting analysis demonstrated that the distribution and expressions of AQP1, AQP2, AQP3, and AQP4 in *L. yarkandensis* kidneys are different. AQP1 was markedly raised in the apical and basolateral plasma membranes of epithelial cells in PT and DTL. The results were in agreement with the findings of AQP1, which is a key protein for water permeability of PT and DTL (Zeidel et al., 1992; Nielsen et al., 1993b; Maeda et al., 1995). In AQP1 null mice, the osmotic water permeability of DTL was markedly decreased by ten-fold compared with that of wild-type mice (Chou et al., 1999). Thus, the higher AQP1 expression in *L. yarkandensis* kidneys might be associated with the rise of water permeability. Our results demonstrated that AQP2, AQP3, and AQP4 were present in principal cells and IMCD cells in the CD, and the final water reabsorption takes place in CD cells through AQP2, AQP3, and AQP4. AQP2 is present on the apical membrane of CD principal cells and acts as a chief target for regulation of CD water permeability by antidiuretic hormone (Kayoko et al., 2010). AQP3 and AQP4 represent the exit pathways of water across the basolateral membrane in the CD principal cells. We also found that AQP2, AQP3, and AQP4 were higher in *L. yarkandensis*, and alterations in their expression may play an important role in increasing the renal concentrating ability. Therefore, the higher expression of AQP1, AQP2, AQP3, and AQP4 in *L. yarkandensis* renal medulla favored for drawing more water from the tubular fluid, producing highly concentrated urine.

Environmental stress can accelerate the evolutionary rate of specific stress-response proteins and generate new functions according to specific environment, enhancing biological adaption to stressful environment (Nei, 2005). For example, cold environment drove the adaptive evolution of leptin of *O. curzoniae* (Yang et al., 2008). Several researchers have reported that the expression of microbial AQPs was correlated with freeze tolerance, deletion of *AQY1* and *AQY2* in a laboratory strain rendered yeast cells that are more sensitive to freezing, while overexpression of these genes improved yeast freeze tolerance (Tanghe et al., 2002, 2005). In addition, some researchers have reported that the expression of plant aquaporins was correlated with drought tolerance. Down-regulation of *PIPs* genes expression in *Lactuca sativa* or *Robinia pseudoacacia* plants, which might be a way to minimize water loss in tissues, and up-regulation of *RpAQP* genes expression, which could be a way to increase water flow to specific tissues during drought stress (Rosa et al., 2006; He et al., 2016). In addition, body water homeostasis is regulated by vasopressin (Fenton and Knepper, 2007), and some investigators have studied the effects of dehydration on vasopressin. For example, in response to dehydration, vasopressin and AQP2 expression in water-deprived rats or desert rodents were increased (Swenson et al., 1997; Gallardo, 2005; Elgot et al., 2017). Desert rodents contain high amounts of vasopressin in the pituitary, plasma and urine (Schmidt-Nielsen and Schmidt-Nielsen, 1952; Mbassa, 1988). In the kidney, vasopressin binds to the vasopressin V2 receptor located at the basolateral plasma membrane, up-regulating the abundance of AQPs and leading to increased water permeability and urinary concentrating capacity (Nielsen et al., 1993a; Terris et al., 1996; Poulsen et al., 2013; Xu and Wang, 2016). Our results indicated that the expression of AQP1, AQP2, AQP3, and AQP4 were higher in *L. yarkandensis* kidneys, this was more likely due to *L. yarkandensis* long-term living in arid environment and possible higher levels of vasopressin. Thus, our results might be correlated with desert animal capacity of drought tolerance.

## Conclusion

**FIGURE 13:**
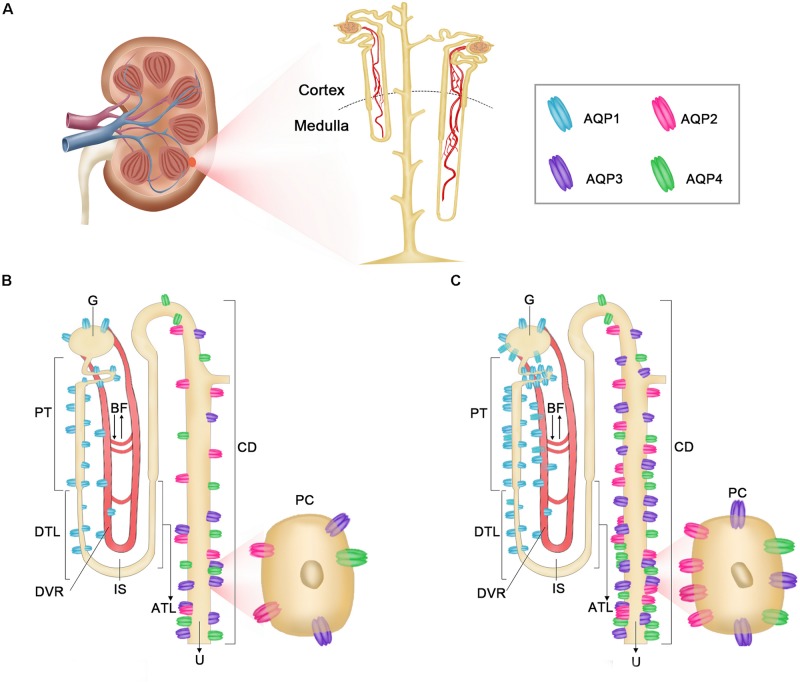
Diagrammatic representation of distribution of AQP1, AQP2, AQP3, and AQP4 in *O. cuniculus* and *L. yarkandensis* kidneys. **(A)** Multipapillary Kidneys anatomy. **(B)** The localization of AQP1, AQP2, AQP3, and AQP4 in *O. cuniculus*. **(C)** The localization of AQP1, AQP2, AQP3, and AQP4 in *L. yarkandensis*. AQP1 (blue) is extremely abundant in the apical and basolateral plasma membrane of G and PCT and PST and DTL cells. AQP2 (pink) is abundant in the apical membrane of principal cells in CD, whereas AQP3 (purple) and AQP4 (green) are both present in the basolateral plasma membrane of the same cells. glomerulus (G), proximal convoluted tubule (PCT), proximal straight tubule (PST), descending thin limbs (DTL), descending vasa recta (DVR), interstitial space (IP), ascending thin limbs (ATL), collecting duct (CD), principal cell (PC), blood flow (BF), urine (U).

In conclusion, the wider medulla and long loop of Henle of *L. yarkandensis* kidneys provides a structural basis for the formation of high osmotic gradient in the medulla, and the osmotic gradient provides the driving force for water reabsorption. The higher expression levels of AQP1, AQP2, AQP3, and AQP4 in the renal medulla of *L. yarkandensis* compared to those in *O. cuniculus* suggested that the renal tubule of *L. yarkandensis* has a rise in water permeability. The cellular distribution of AQP1, AQP2, AQP3, and AQP4 in *L. yarkandensis* and *O. cuniculus* kidneys was summarized in Figure 13. Blood is filtered at the glomerular region and is then passed into the lumen of the proximal tubule. Most of this filtrate is resorbed through AQP1 in PT and DTL epithelial cells. The water is transported to the vasa recta to maintain hypertonicity in the interstitial space through AQP1 in the endothelial cells of DVR. The final concentration of urine is determined by AQP2 in the apical membrane of CD principal cells and IMCD cells, and a combination of AQP3 and AQP4 in the basolateral membrane of CD principal cells and IMCD cells. The higher expression levels of AQP1, AQP2, AQP3, and AQP4 proteins in *L. yarkandensis* kidneys maybe enhance water reabsorption, maintaining body water.

## Data Availability

All datasets generated for this study are included in the manuscript and/or the Supplementary Files.

## Ethics Statement

All animal procedures were approved by the Animal Care and Use Committee of Hubei Province and Xinjiang Uygur Autonomous Region, China, and were conducted in accordance with the guidelines developed by the China Council on Animal Care and Protocol.

## Author Contributions

GL designed and wrote the manuscript. JZ designed and wrote the manuscript and carried out the data analysis, Quantitative RT-PCR, western blot, and immunohistochemical studies. SL, FD, and BB did the sample collection and staining. SH and BW did the sample collection and processing.

## Conflict of Interest Statement

The authors declare that the research was conducted in the absence of any commercial or financial relationships that could be construed as a potential conflict of interest.
