# Supplementary material for: Higher Expression Levels of Aquaporin Family of Proteins in the Kidneys of Arid-Desert Living Lepus yarkandensis
Source: Front Physiol. 2019 Sep 12;10:1172. doi: 10.3389/fphys.2019.01172 (PMC6751383; doi:10.3389/fphys.2019.01172)
Supplement: Supplementary file 1 [file Data_Sheet_1.PDF]

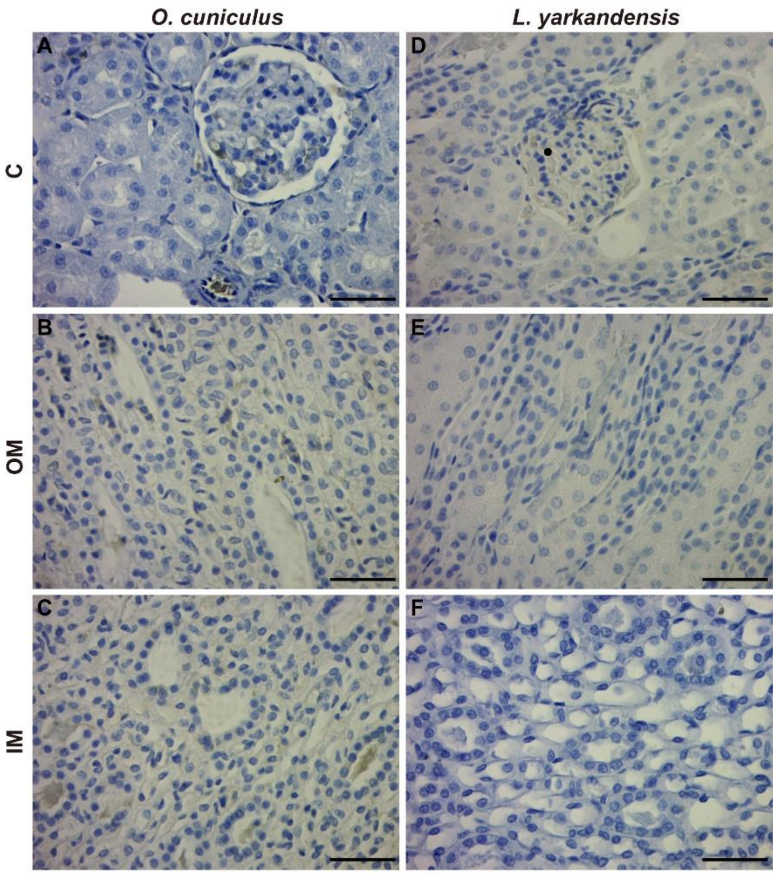

FIGURE S1 | The negative control slides included renal C, OM and IM of a tissue section of *O. cuniculus* (A-C) and *L. yarkandensis* (D-F) in the immunohistochemistry experiment. scale bar 50 μm. cortex (C), outer medulla (OM), inner medulla (IM).
